# Urinary cMet as a prognostic marker in immunoglobulin A nephropathy

**DOI:** 10.1111/jcmm.15636

**Published:** 2020-08-21

**Authors:** Jung Nam An, Lilin Li, Junghun Lee, Seung‐Shin Yu, Jin Hyuk Kim, Jeonghwan Lee, Yong Chul Kim, Dong Ki Kim, Yun Kyu Oh, Chun Soo Lim, Yon Su Kim, Sunyoung Kim, Seung Hee Yang, Jung Pyo Lee

**Affiliations:** ^1^ Department of Internal Medicine Hallym University Sacred Heart Hospital Anyang Korea; ^2^ Department of Internal Medicine Seoul National University College of Medicine Seoul Korea; ^3^ Department of Intensive Care Unit Yanbian University Hospital Jilin China; ^4^ R&D Center for Innovative Medicines Helixmith Co., Ltd. Seoul Korea; ^5^ Department of Internal Medicine Seoul National University Boramae Medical Center Seoul Korea; ^6^ Department of Internal Medicine Seoul National University Hospital Seoul Korea; ^7^ Seoul National University Kidney Research Institute Seoul Korea; ^8^ Biomedical Research Institute Seoul National University Hospital Seoul Korea

**Keywords:** cMet agonistic antibody, complete remission, immunoglobulin A nephropathy, inflammation, mesangial cells, prognostic marker, proliferation, proteinuria, urinary cMet

## Abstract

The prediction of prognosis in patients with immunoglobulin A nephropathy (IgAN) is challenging. We investigated the correlation between urinary cMet (ucMet) levels and clinical parameters and examined the effects of cMet agonistic antibody (cMet Ab) in an in vitro IgAN model. Patients diagnosed with IgAN (n = 194) were divided into three groups representing undetectable (Group 1), below‐median (Group 2) and above‐median (Group 3) levels of ucMet/creatinine (ucMet/Cr). Stained kidney biopsy samples were graded according to cMet intensity. Primary‐cultured human mesangial cells were stimulated with recombinant tumour necrosis factor (TNF)‐α and treated with cMet Ab. Our results showed that ucMet/Cr levels positively correlated with proteinuria (*P* < .001). During the follow‐up, patients in Group 3 showed a significantly lower probability of complete remission (CR; uPCr < 300 mg/g) than those in groups 1 and 2, after adjusting for blood pressure, estimated glomerular filtration rate, and proteinuria, which influence clinical prognosis (HR 0.60, *P* = .038); moreover, ucMet/Cr levels were also associated with glomerular cMet expression. After TNF‐α treatment, the proliferation of mesangial cells and increased interleukin‐8 and intercellular adhesion molecule‐1 expression were markedly reduced by cMet Ab in vitro. In conclusion, ucMet/Cr levels significantly correlated with proteinuria, glomerular cMet expression, and the probability of CR. Further, cMet Ab treatment alleviated the inflammation and proliferation of mesangial cells. Hence, ucMet could serve as a clinically significant marker for treating IgAN.

## 1 INTRODUCTION

Immunoglobulin A nephropathy (IgAN) is the most common form of primary glomerulonephritis,^1^, ^2^, ^3^, ^4^ occurring in approximately 40% of Korean adults.^5^, ^6^ Deposition of IgA‐containing immunocomplexes in the glomerular mesangium results in mesangial hypercellularity, mesangial expansion, glomerular inflammation and structural changes. IgAN is a common cause of chronic kidney disease and end‐stage renal disease (ESRD) worldwide.^1^, ^7^, ^8^, ^9^ Approximately 25%‐30% of patients with IgAN progress to ESRD, around 20‐25 years after initial diagnosis.^10^, ^11^

The clinical features and natural progression of IgAN are diverse,^7^, ^12^, ^13^ and only minor haematuria may be seen throughout the patient's lifetime, sometimes with proteinuria. However, rapid decline of renal function may lead to ESRD after diagnosis. Hence, it is important for nephrologists to identify and prioritize high‐risk patients with poor prognosis. The established prognostic factors of IgAN currently include proteinuria, blood pressure, renal function and biopsy findings at the time of diagnosis.^12^, ^13^, ^14^, ^15^, ^16^, ^17^, ^18^ The development of methods to predict disease prognosis without invasive renal biopsy has been clinically challenging. Among the prognostic tools employed, large amounts of urine samples may be easily, simply and repeatedly obtained by a non‐invasive method; therefore, urine samples have been used to actively identify novel biomarkers.

cMet, a transmembrane tyrosine kinase receptor of hepatocyte growth factor (HGF), is involved in cell growth, survival and regeneration.^19^, ^20^ The HGF/cMet pathway regulates the progression of various diseases by reducing oxidative stress, inflammation, apoptosis and fibrosis.^21^, ^22^ The role of urinary cMet (ucMet) as a biomarker in diabetic nephropathy has recently been identified.^23^ In addition, animal and cell experiments have demonstrated the effects of attenuating kidney fibrosis and acute kidney injury (AKI) using agonistic monoclonal antibodies of cMet (cMet Ab).^23^, ^24^, ^25^ In glomerular endothelial cells (GECs) and proximal tubular epithelial cells (PTECs), cell markers and the expression region of cMet are well merged.^23^, ^24^ However, the application of cMet as a clinical marker of IgAN, and the ability of cMet Ab to ameliorate IgAN, is yet to be explored.

Therefore, in the present study, we investigated the correlation between ucMet levels at the time of diagnosis and clinical manifestations in IgAN, along with the effects of ucMet levels on clinical outcomes. Furthermore, we verified if treatment with cMet Ab reduced inflammation and mesangial proliferation in in vitro IgAN models.

## 2 MATERIALS AND METHODS

### 2.1 Study population and data collection

The present study was approved by the Institutional Review Board of Seoul National University Boramae Medical Center (no. 10‐2019‐27/043). Informed consent was obtained from all patients prior to the use of urine and kidney tissue samples. All clinical investigations were conducted in accordance with the guidelines of the 2013 Declaration of Helsinki.

Patients diagnosed with IgAN, confirmed via kidney biopsy, from April 2011 to March 2020 at the Seoul National University Boramae Medical Center and Seoul National University Hospital were enrolled in the present study. Urine samples collected from 194 patients were analysed in this investigation.

Demographic and clinical characteristics at the time of kidney biopsy, including comorbidities, blood pressure, serum creatinine (sCr), estimated glomerular filtration rate (eGFR), spot urine protein‐to‐creatinine ratio (uPCr) and other laboratory findings (serum albumin, uric acid, total cholesterol and IgA), were collected from electronic medical records. eGFR was calculated using isotope dilution mass spectrometry and a traceable, modified, Modification of Diet in Renal Disease equation. The use of therapeutic agents post‐diagnosis, including angiotensin‐converting‐enzyme inhibitors or angiotensin II receptor blockers, statins and immunosuppressive agents, was also investigated.

Kidney tissue samples were evaluated by light, electron and immunofluorescence microscopy and diagnosed by a renal pathologist. Interstitial fibrosis, tubular atrophy and interstitial inflammation were scored based on the percentage of affected area as follows: 0, none; 1, mild, ≤25%; 2, moderate, 26%‐50%; and 3, severe, >50%. Fibrointimal thickening and hyaline arteriolosclerosis were also evaluated.

### 2.2 Measurement of urine soluble cMet levels

ucMet levels were measured using an enzyme‐linked immunosorbent assay (KHO 2031; Thermo Fisher Scientific Inc) according to the manufacturer's instructions. All measurements were performed in a blinded manner, and in duplicate. Sample urine creatinine levels were measured (Roche C702, CREJ2), adjusted, and expressed as the ucMet‐to‐urine Cr ratio (ucMet/Cr). The association between the ucMet/Cr levels and several laboratory and histological findings was analysed.

### 2.3 Clinical outcomes

Patients were divided into three groups based on the ucMet/Cr levels as follows: Group 1, undetectable levels of ucMet; Group 2, below‐median levels of ucMet/Cr; and Group 3, above‐median levels of ucMet/Cr Then, we analysed the correlation between ucMet/Cr and clinical outcome in the three groups of the patients with IgAN who were followed up for at least 3 months after the biopsy was performed. In the present study, clinical outcome was defined as complete remission (CR) following the KDIGO guideline, uPCr < 300 mg/g.

### 2.4 Immunohistochemistry of kidney biopsy samples

Unstained slides of the tissue samples obtained from the study population were used. Paraffin‐embedded kidney tissue samples were cut into 4‐μm‐thick sections, deparaffinized, and rehydrated using xylene and ethanol. After blocking the endogenous streptavidin activity using 3% hydrogen peroxide, the sections were stained with anti‐cMet antibody (1:200; ab51067, Abcam) and incubated at 4°C overnight. Next, samples were incubated with dextran polymer conjugated with horseradish peroxidase (GBI Labs) for 5 minutes at room temperature. Finally, all sections were counterstained with Mayer's haematoxylin (ScyTek Laboratories) and examined by light microscopy. In each slide, a minimum of 10 fields were assessed for glomeruli (at 400×) and tubules (at 200×), in a blinded fashion by a kidney pathologist. The cMet intensity score was graded semi‐quantitatively from 0 to 3 as follows: 0, absence of or faint staining; 1, mild staining; 2, moderate staining; and 3, strong staining.

### 2.5 In vitro IgAN model

Primary‐cultured human mesangial cells were used in the in vitro model of IgAN. The Institutional Review Board of Seoul National University Hospital (No. 1404‐117‐515) approved the protocols to obtain normal tissue specimens of the resected kidneys from patients with renal cell carcinoma. As previously reported,^26^ mesangial cells were isolated from the glomeruli using a differential sieving technique. The glomerular fraction was concentrated to at least 95% by centrifugation. The isolated cells were incubated in Dulbecco's modified Eagle's medium supplemented with 10 mmol/L D‐glucose, 15% foetal bovine serum, 100 U/mL penicillin, 100 mg/mL streptomycin and 2 mmol/L glutamine, and passaged every 72 hours.

After 24 hours of incubation in serum‐free media, the cells were stimulated with recombinant human tumour necrosis factor (TNF)‐α (10 ng/mL; R&D Systems) and treated with cMet Abs (0.5 and 1.0 μg/mL; Helixmith Co., Ltd.) for 24 hours. Human IgG (1.0 μg/mL, R&D Systems) was also used. The cMet Abs concentration used in this study was similar to previous reports.^23^, ^24^, ^25^ Mesangial cell proliferation was quantified using a colorimetric MTS cell proliferation assay kit (Promega) according to the manufacturer's protocols.

### 2.6 Immunofluorescence staining

Kidney sections were probed with immunofluorescence antibodies against desmin, a marker of mesangial cells (MA5‐13259; Thermo Fisher Scientific Inc), and cMet (ab216574; Abcam) in a blocking reagent overnight at 4°C. A secondary Alexa Fluor^®^ 647‐conjugated goat anti‐mouse antibody and Alexa Fluor^®^ 488‐conjugated goat anti‐rabbit antibody (Molecular Probes) were incubated, respectively, for 1 hour at 22‐25°C. All sections were washed and incubated for an additional 5 minutes with 4′,6‐diamidino‐2‐phenylindole (DAPI; Molecular Probes) for counterstaining. For negative controls, the primary antibodies were omitted.

After 24 hours of stimulation, the mesangial cells were harvested and stained with immunofluorescence antibodies against Ki‐67 and Intercellular Adhesion Molecule (ICAM)‐1 (Abcam) in a blocking reagent overnight at 4°C. Alexa Fluor^®^ 647‐conjugated goat anti‐rabbit antibody and Alexa Fluor^®^ 488‐conjugated goat anti‐mouse antibody (Molecular Probes) were incubated for 1 hour at 22‐25°C, respectively. To counterstain the negative controls, DAPI (Molecular Probes) was used and primary antibodies were omitted. Confocal microscopic examination was performed in a blind and random manner, and images were captured using Leica TCS SP8 STED CW (20X/0.7 NA objective lens of the DMI 6000 inverted microscope; Leica, Mannheim, Germany) and MetaMorph version 7.8.10 software (Universal Imaging).

### 2.7 Flow cytometry

For intracellular staining, cells treated with human Fc Block anti‐CD16/32 (BD Biosciences) were stained with allophycocyanin‐conjugated anti‐IL‐8 (BD Biosciences) or with the isotype control for 1 hour. IL‐8^+^ mesangial cells were measured and analysed with a BD FACS Canto platform and BD FACS Diva version 8.0 (BD Biosciences).

### 2.8 Statistical analysis

Categorical variables, described as frequencies and proportions, were compared using chi‐squared tests. Continuous variables were expressed as mean ± standard deviation, or standard error of the mean where appropriate, and were compared using either the Student's *t* test or one‐way analysis of variance test. Non‐normally distributed variables were expressed as medians with an interquartile range and were compared using the Mann‐Whitney *U* test or Kruskal‐Wallis test. Pearson correlation coefficients were determined to explore the linear relationship between ucMet/Cr levels and various clinical parameters. Cox proportional hazard models were used to assess the correlation between ucMet/Cr levels and clinical outcomes. Statistical analyses were performed using SPSS version 22 (IBM software, USA) and GraphPad Prism 8.0 (GraphPad Software, Inc). Statistical significance was determined at *P < *.05.

## 3 RESULTS

### 3.1 Baseline characteristics and histologic findings by ucMet group

The baseline characteristics of each of the three groups divided by ucMet/Cr levels are described in Table 1 (n = 194). Diabetes, hypertension, body mass index, renal pathological findings, IgA levels, microscopic haematuria and post‐diagnosis medications did not show any significant differences between the three groups. However, compared to the patients in Group 2, those in Group 3 were older and showed lower serum albumin levels and higher uPCr. No significant difference in sCr and eGFR was observed between these two groups.

**Table 1:** Baseline characteristics and demographics based on the urine cMet/creatinine level

	Total number of patients (n = 194)	Group 1 (n = 38; undetectable levels of cMet/Cr)	Group 2 (n = 77; cMet/Cr < 0.0121)	Group 3 (n = 79; cMet/Cr ≥ 0.0121)	*P* value
Age (y)	41 (28, 54)	46 (29, 55)	35 (25, 45)	43 (31, 58)	.005
Male	105 (54.1)	21 (55.3)	53 (68.8)	31 (39.2)	.001
History of smoking	34 (17.5)	4 (10.5)	18 (23.4)	12 (15.2)	.182
Diabetes Mellitus	8 (4.1)	3 (7.9)	1 (1.3)	4 (5.1)	.739
Hypertension	106 (54.6)	15 (39.5)	41 (53.2)	50 (63.3)	.050
Systolic blood pressure (mm Hg)	127.3 ± 18.8	122.6 ± 15.8	128.6 ± 19.1	128.3 ± 19.7	.233
Diastolic blood pressure (mm Hg)	79.6 ± 14.0	76.4 ± 11.2	80.4 ± 13.5	80.5 ± 15.6	.278
Body mass index (kg/m^2^)	23.9 ± 3.4	24.4 ± 3.7	23.7 ± 3.3	23.9 ± 3.3	.573
Microscopic haematuria	177 (91.2)	34 (89.5)	69 (89.6)	74 (93.7)	.610
SMK Lee grade					
I	10 (5.2)	3 (7.9)	5 (6.5)	2 (2.5)	.039
II	96 (49.5)	22 (57.9)	40 (51.9)	34 (43.0)	
III	46 (23.7)	9 (23.7)	17 (22.1)	20 (25.3)	
IV	14 (7.2)	1 (2.6)	6 (7.8)	7 (8.9)	
V	7 (3.6)	0 (0.0)	4 (5.2)	3 (3.8)	
Haas Class					
I	6 (3.1)	2 (2.6)	3 (3.9)	1 (1.3)	.117
II	18 (9.3)	4 (5.2)	7 (9.1)	7 (8.9)	
III	80 (41.2)	18 (47.4)	35 (45.5)	27 (34.2)	
IV	50 (25.8)	9 (23.7)	18 (23.4)	23 (29.1)	
V	18 (9.3)	2 (2.6)	8 (10.4)	8 (10.1)	
VI	1 (0.5)	0 (0.0)	1 (1.3)	0 (0.0)	
Mesangial hypercellularity	185 (95.4)	37 (97.4)	74 (96.1)	74 (93.7)	.339
Interstitial fibrosis/tubular atrophy	168 (86.6)	37 (97.4)	65 (84.4)	66 (83.5)	.093
Moderate to severe	41 (21.1)	9 (23.7)	16 (20.8)	16 (20.3)	.353
Interstitial inflammation	155 (79.9)	30 (78.9)	59 (76.6)	66 (83.5)	.552
Moderate to severe	33 (17.0)	5 (13.2)	15 (19.5)	13 (16.5)	.790
Vessel					
Fibrointimal thickening	70 (36.1)	12 (31.6)	24 (31.2)	34 (43.0)	.247
Hyaline arteriolosclerosis	30 (15.5)	7 (18.4)	10 (13.0)	13 (16.5)	.714
Global sclerosis (%)	16.7 (4.5, 34.2)	16.9 (9.2, 33.7)	14.3 (5.2, 37.3)	15.4 (0.0, 30.0)	.376
Segmental sclerosis (%)	3.3 (0.0, 12.1)	1.2 (0.0, 11.8)	3.6 (0.0, 11.1)	3.7 (0.0, 12.5)	.808
Crescent (%)	0.0 (0.0, 0.0)	0.0 (0.0, 0.0)	0.0 (0.0, 0.0)	0.0 (0.0, 0.0)	.396
Laboratory findings					
Serum creatinine (sCr) (mg/dL)	1.00 (0.79, 1.46)	1.24 (0.90, 1.56)	0.98 (0.81, 1.47)	1.00 (0.75, 1.33)	.154
Estimated GFR (mL/min/1.73 m^2^)	69.3 (47.5, 102.2)	57.5 (45.8, 86.7)	80.3 (48.2, 104.5)	67.3 (49.7, 104.8)	.129
Urine protein/creatinine ratio (mg/mgCr)	1.53 (0.70, 2.77)	1.17 (0.60, 2.33)	1.26 (0.34, 2.13)	2.21 (1.01, 4.16)	<.001
Immunoglobulin A (mg/dL)	322.0 (251.0, 415.0)	322.0 (236.0, 415.5)	305.0 (247.3, 410.8)	344.0 (262.3, 417.8)	.348
Albumin (g/dL)	3.8 (3.5, 4.1)	4.0 (3.7, 4.2)	4.0 (3.6, 4.2)	3.7 (3.4, 3.9)	<.001
hs‐CRP (mg/dL)	0.10 (0.03, 0.30)	0.06 (0.03, 0.16)	0.10 (0.05, 0.29)	0.12 (0.02, 0.39)	.271
Total cholesterol (mg/dL)	182.5 (158.0, 216.5)	175.0 (158.0, 223.0)	176.0 (150.5, 222.0)	188.0 (159.5, 212.0)	.784
Uric acid (mg/dL)	6.2 (5.2, 7.5)	6.9 (5.7, 8.4)	6.1 (5.4, 7.5)	6.0 (4.6, 7.5)	.105
Urine cMet (ng/mL)	0.75 (0.09, 1.84)	—	0.41 (0.16, 0.93)	1.90 (1.36, 3.17)	<.001
Urine cMet/Cr (ng/mgCr)	0.009 (0.001, 0.020)	—	0.003 (0.001, 0.008)	0.022 (0.017, 0.038)	<.001
Treated with RAS blockade	117 (60.3)	21 (55.3)	41 (53.2)	55 (69.6)	.088
Treated with statin	60 (30.9)	11 (28.9)	21 (27.3)	28 (35.4)	.521
Treated with immunosuppressive agents	29 (14.9)	3 (7.9)	10 (13.0)	16 (20.3)	.177

Note

The data are expressed as the proportion (%), mean ± SD or median (IQR).

Abbreviations: GFR, glomerular filtration rate; hs‐CRP, high‐sensitivity C‐reactive protein; RAS, renin‐angiotensin system.

**Figure 1:**
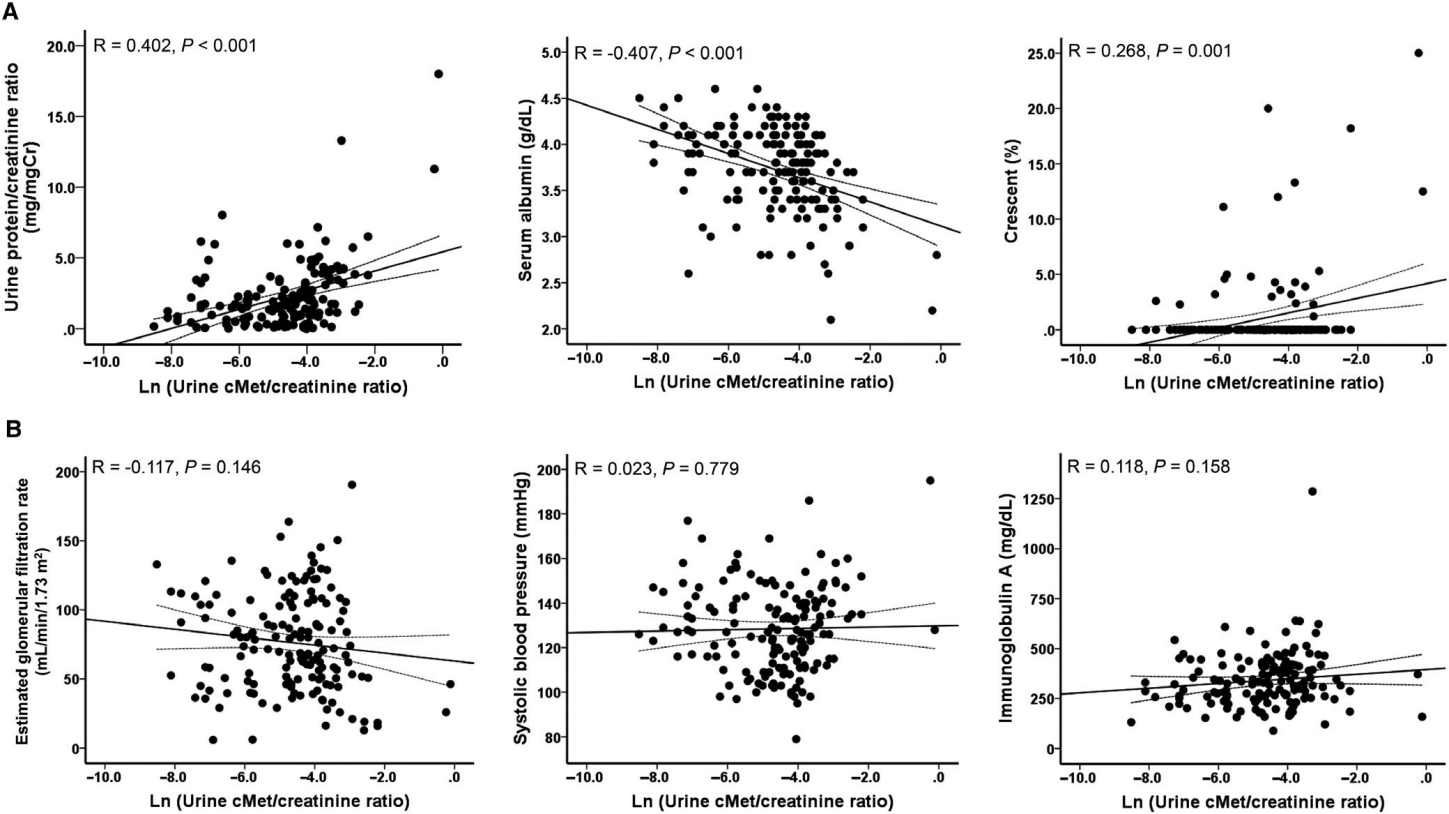
The association between ucMet/Cr levels and clinical parameters. A, The ucMet/Cr level converted to natural logarithm is positively correlated with proteinuria (Pearson correlation coefficients; *R* = .402, *P* < .001) and the percentage of crescent (Pearson correlation coefficients; *R* = .268, *P* = .001), and negatively correlated with serum albumin level (Pearson correlation coefficients; *R* = −.407, *P* < .001). B, No correlation was seen between ucMet/Cr levels and eGFR, IgA, and systolic blood pressure

Of 156 patients, the measured ucMet/Cr level was converted into a natural logarithm (Figure 1) to investigate the correlation with the laboratory results. The ucMet/Cr level was not significantly correlated with eGFR at the time of diagnosis, but was positively correlated with uPCr (*R* = .402, *P* < .001) and negatively correlated with the serum albumin level (*R* = −.407, *P* < .001). In addition, there was no association between ucMet/Cr level and IgA levels and systolic blood pressure. Among the pathological results, only a weak positive correlation was found with crescent (*R* = .268, *P* = .001).

### 3.2 Renal outcomes

**Figure 2:**
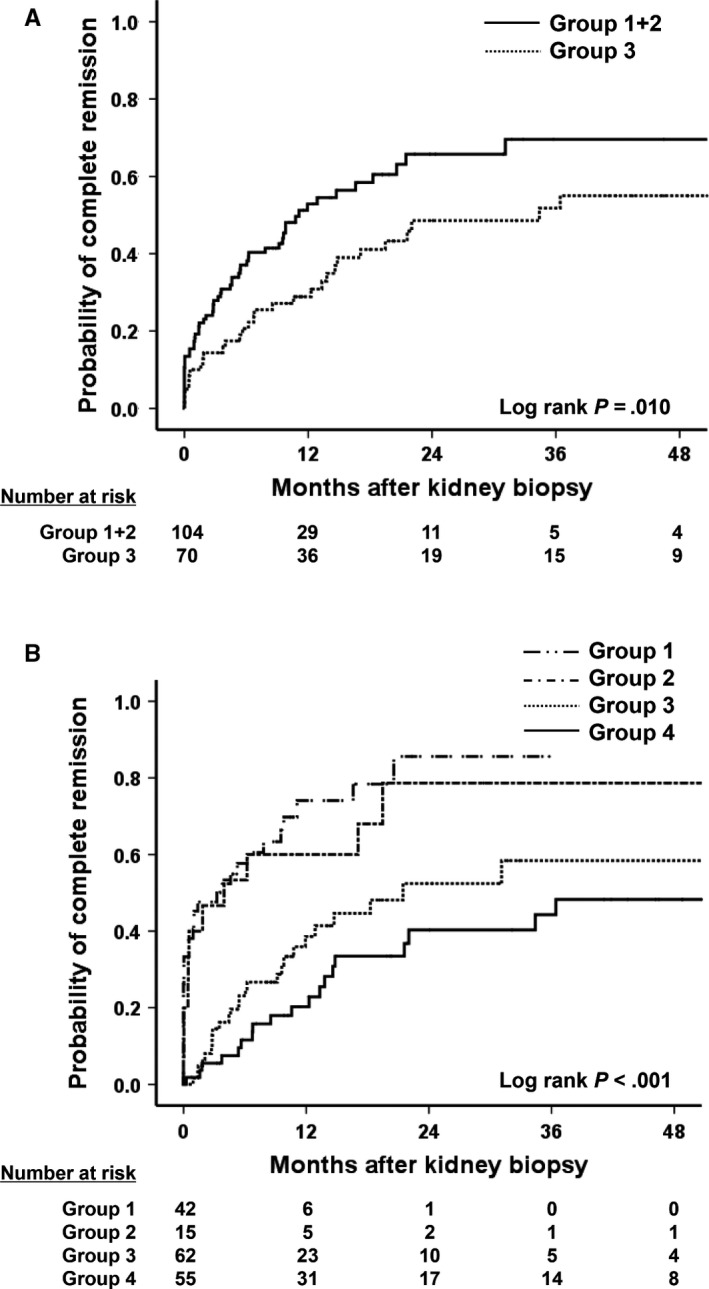
Comparison of the CR probability according to ucMet/Cr level. A, Patients in Group 3 had a significantly lower probability of reaching CR compared to patients in Groups 1 + 2 (Log rank *P* = .010). B, Four combinations of proteinuria and ucMet/Cr levels were made, and the probabilities of reaching CR were compared. In patients with proteinuria >1 g/d, higher ucMet/Cr levels resulted in lower CR (Log rank *P* < .001)

The hazard ratios (HRs) of CR between Groups 1 + 2 and Group 3 patients who were observed for at least 3 months after diagnosis of IgAN (n = 174) were assessed. During the median 15‐month follow‐up, patients in Group 3 had a significantly lower probability of CR compared to patients in Groups 1 + 2 (Table 2; Figure 2A). This result was also significant after adjusting for factors such as systolic BP, eGFR and proteinuria, which influence clinical prognoses (model 2; HR 0.60, 95% CI 0.37‐0.97, *P* = .038).

**Table 2:** The effect of urine cMet/Cr level on the probability of complete remission

	Unadjusted		Model 1		Model 2		Model 3		Model 4	
	HR (95% CI)	*P* value	aHR (95% CI)	*P* value	aHR (95% CI)	*P* value	aHR (95% CI)	*P* value	aHR (95% CI)	*P* value
Groups 1 + 2	Reference		Reference		Reference		Reference		Reference	
Group 3	0.56 (0.36‐0.88)	.011	0.51 (0.32‐0.80)	.004	0.60 (0.37‐0.97)	.038	0.53 (0.34‐0.84)	.007	0.63 (0.39‐1.02)	.058

Abbreviations: aHR, adjusted hazard ratio; CI, confidence interval.

Model 1: adjusted for systolic blood pressure and eGFR.

Model 2: adjusted for proteinuria in addition to Model 1.

Model 3: adjusted for the administration of RAS blockers or immunosuppressants in addition to Model 1.

Model 4: adjusted for the administration of RAS blockers or immunosuppressants in addition to Model 2.

Four combinations of uPCr and ucMet/Cr levels were made at the point of diagnosis. In groups 1 to 4, it was noted that patients were progressively older, with increased blood pressure, poorer renal pathology and renal function, raised levels of total cholesterol, and decreased levels of serum albumin (Table S1). In addition, many patients were treated with renin‐angiotensin system blockers and immunosuppressive agents. Compared to patients with proteinuria <1 g/d and undetectable or low ucMet/Cr levels, increasing ucMet/Cr levels did not affect the probability of CR (model 2; Group 1 vs 2, HR 0.61, 95% CI 0.29‐1.29, *P* = .194) (Table 3; Figure 2B). Conversely, although ucMet/Cr levels were low or undetected, patients with proteinuria ≥1 g/d were 63% less likely to develop CR (model 2; Group 1 vs 3). An increase in the ucMet/Cr level further decreased the probability of CR (model 2; Group 1 vs 4, HR 0.23, 95% CI 0.12‐0.43, *P* < .001). Results were statistically significant even when the use of therapeutic agents was adjusted (model 4; Group 1 vs 4, HR 0.25, 95% CI 0.13‐0.47, *P* < .001).

**Table 3:** The effect of urine cMet/Cr level and proteinuria on the probability of complete remission

Group	Urine cMet/Cr	uPCr	Unadjusted		Model 1		Model 2		Model 3		Model 4	
			HR (95% CI)	*P*	aHR (95% CI)	*P*	aHR (95% CI)	*P*	aHR (95% CI)	*P*	aHR (95% CI)	*P*
1	Undetectable + low	<1 g/d	Reference		Reference		Reference		Reference		Reference	
2	High	<1 g/d	0.73 (0.37‐1.47)	.380	0.62 (0.30‐1.27)	.188	0.61 (0.29‐1.29)	.194	0.65 (0.32‐1.34)	.244	0.65 (0.30‐1.38)	.261
3	Undetectable + low	≥1 g/d	0.33 (0.19‐0.55)	<.001	0.36 (0.21‐0.62)	<.001	0.37 (0.21‐0.65)	.001	0.38 (0.22‐0.66)	.001	0.40 (0.22‐0.70)	.001
4	High	≥1 g/d	0.21 (0.12‐0.37)	<.001	0.22 (0.12‐0.40)	<.001	0.23 (0.12‐0.43)	<.001	0.24 (0.13‐0.43)	<.001	0.25 (0.13‐0.47)	<.001

Abbreviations: aHR, adjusted hazard ratio; CI, confidence interval; Cr, creatinine; uPCr, urine protein/creatinine ratio.

Model 1: adjusted for systolic blood pressure and eGFR.

Model 2: adjusted for sex and age in addition to Model 1.

Model 3: adjusted for the administration of RAS blockers or immunosuppressants in addition to Model 1.

Model 4: adjusted for the administration of RAS blockers or immunosuppressants in addition to Model 2.

### 3.3 cMet expression in kidney tissues and ucMet/Cr

**Figure 3:**
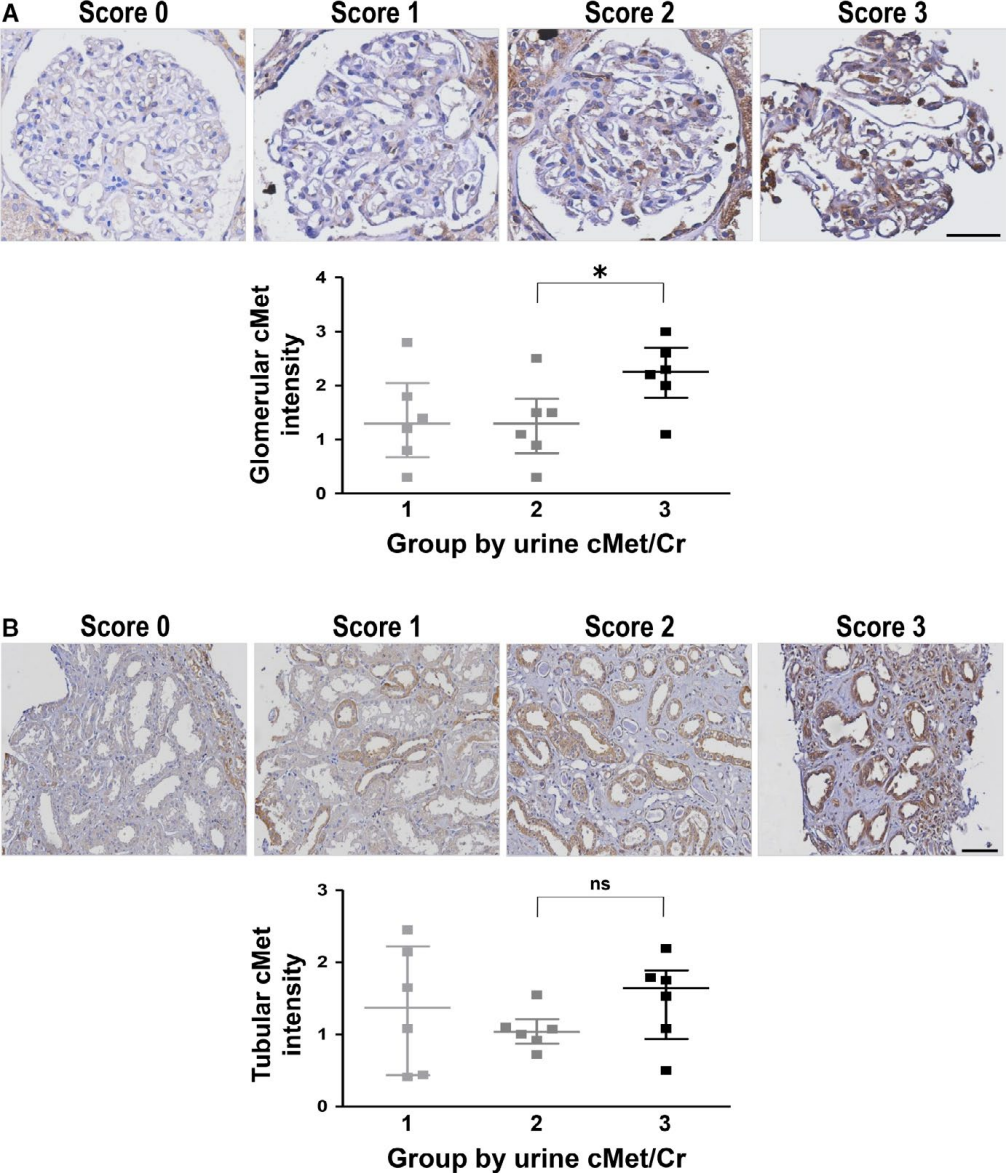
cMet intensity in the kidney tissue and ucMet/Cr level. A, Glomerular cMet expression was correlated with ucMet/Cr level, B, but tubular cMet expression was not. Magnification: 600× (bar = 50 μm; glomerulus), 200× (bar = 100 μm; tubule). All data are presented as the mean ± SEM. *P < .05 (unpaired *t* test)

cMet staining in human kidney tissues (n = 18) and representative images according to the quantified scores are shown in Figure 3. After measuring and scoring the cMet intensity in the glomeruli, the results were divided based on the ucMet/Cr levels of the patients. It was seen that as ucMet/Cr levels increased, Met intensity in the glomeruli also increased (Figure 3A). On the other hand, Met intensity in the tubules was not correlated with ucMet/Cr levels (Figure 3B).

### 3.4 In vitro model for IgAN

**Figure 4:**
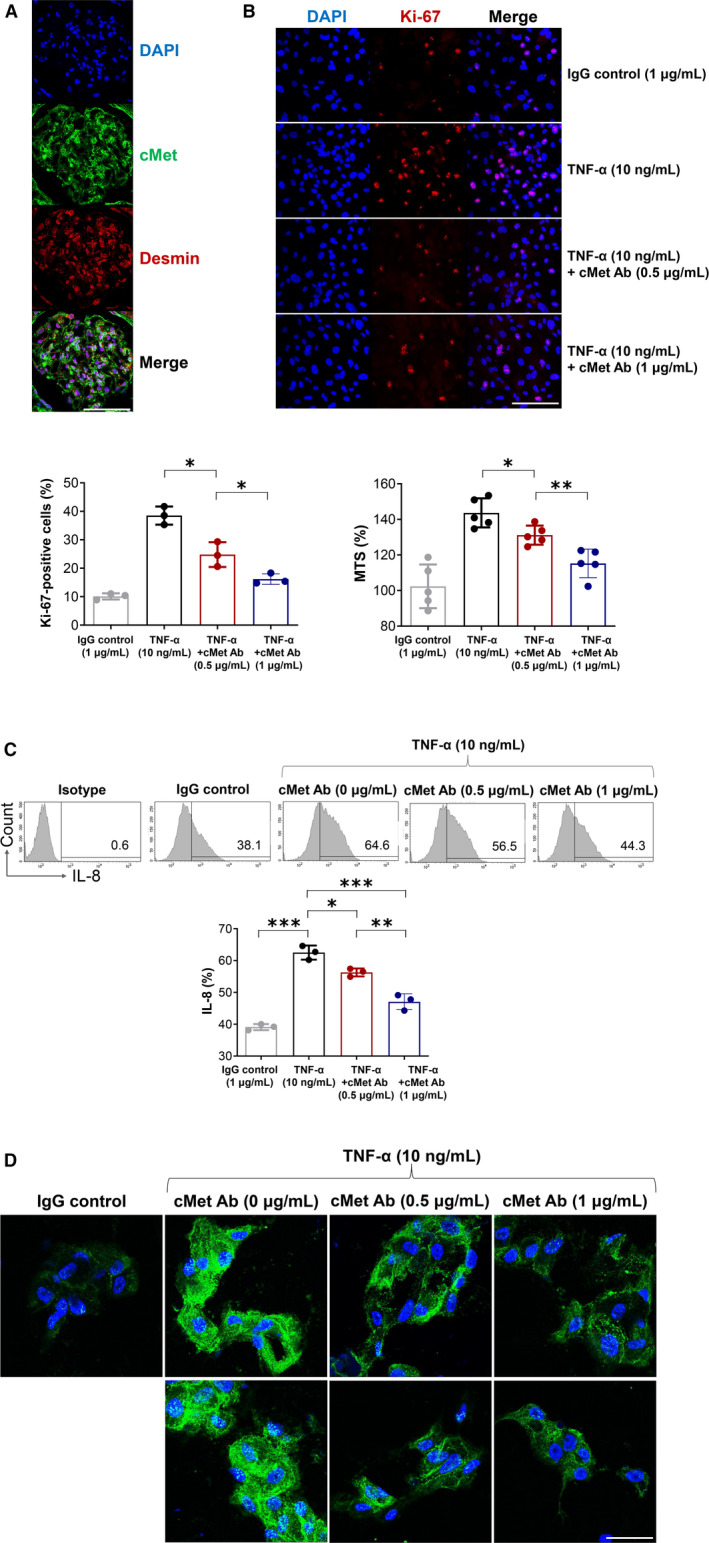
Proliferation and inflammation of mesangial cells alleviated by cMet Ab treatment. A, Observation of cMet and desmin (a mesangial cells marker) expressions which are confirmed to merge well at the same site. Magnification: 800× (bar = 50 μm). B, Ki‐67‐positive cell expression and proliferation were increased after TNF‐α stimulation and decreased dose‐dependently after cMet Ab treatment. The data shown are representative of three independent experiments (N = 3/group; N = 5/group). Magnification: 400× (bar = 100 μm). C, Flow cytometry showed that the IL‐8^+^ cells increased by approximately 1.7 times by TNF‐α stimulation compared to the control. A dose‐dependent decrease was observed on treatment with cMet Ab (N = 3/group). D, ICAM‐1 expression was also increased on TNF‐α stimulation; it was seen to reduce after cMet Ab treatment. Magnification: 800× (bar = 50 μm). The data shown are representative of three independent experiments. All data are presented as the mean ± SEM. *P < .05 (unpaired *t* test); **P < .01 (unpaired *t* test); ***P < .001 (unpaired *t* test)

First, we identified mesangial cells using desmin staining in a kidney biopsy slide sample of an actual IgAN patient and then stained cMet, which confirmed that the mesangial proliferation and expansion and cMet expression were merged well (Figure 4A).

Next, mesangial cells were treated with TNF‐α in a primary culture and observed for 24 hours. Their proliferation was confirmed by Ki‐67 expression and the MTS assay. After treatment with cMet Ab, the number of Ki‐67‐positive cells decreased in a dose‐dependent manner. The MTS assay also confirmed that the proliferation of mesangial cells decreased significantly (Figure 4B). The expression of IL‐8^+^ cells increased approximately 1.7‐fold after stimulation with TNF‐α. This was reduced dose‐dependently after treatment with cMet Ab. When treated with cMet Ab at a concentration of 1 μg/mL, IL‐8^+^ cell expression was similar to that of the control (Figure 4C). We then analysed the expression of ICAM‐1, involved in the progression of various types of glomerulonephritis, by leukocyte infiltration and macrophage accumulation. ICAM‐1 expression increased with TNF‐α stimulation in mesangial cells; however, this expression was significantly decreased on treatment with cMet Ab (Figure 4D).

## 4 DISCUSSION

cMet levels, measured in urine samples during the diagnosis of IgAN, were significantly correlated with proteinuria, serum albumin levels and crescent findings. During the follow‐up period, the probability of CR decreased significantly in the patients in whom ucMet/Cr levels were above the median value compared to those in whom ucMet/Cr levels were undetected or below the median value. Glomerular cMet expression was positively correlated with ucMet/Cr levels, but this was not observed with the tubular cMet expression. However, TNF‐α induced inflammation and proliferation in mesangial cells. When treated with cMet Ab, mesangial cell proliferation and IL‐8 and ICAM‐1 expression were significantly ameliorated.

Recent studies have reported that several markers in serum and urine samples, such as angiotensinogen,^27^ epidermal growth factor,^28^ fibroblast growth factor‐23,^29^ galactose‐deficient IgA1,^30^ autoantibodies against galactose‐deficient IgA1,^31^ and kidney injury molecule‐1,^32^ can be used to predict the early progression of IgAN. Recently, matrix metalloproteinase‐7 levels in urine samples have been shown to predict the progression of IgAN in addition to existing histopathological scores and clinical information.^33^ However, these studies were performed in a relatively small number of patients and did not confirm the expression of markers in the kidney tissue, and the correlation with the level of markers. In addition, the mechanism of action of the markers was not proven.

In the present study, treatment with TNF‐α imparted a proliferative and inflammatory phenotype to human mesangial cells and this effect was ameliorated by cMet Ab treatment. ICAM‐1 expression is stimulated and increased by oxidative stress and TNF‐α and is associated with disease progression in several types of human and experimental glomerulonephritis.^34^, ^35^ Further, IL‐8 is a key mediator associated with inflammation^36^ and cMet Ab significantly reduced its expression, thereby alleviating IgAN.

Soluble cMet plays a role in various diseases. It was reported to be a major marker of cancer progression,^37^ including cell migration, and its expression contributed to the protection and recovery of endothelial cells after injury in severe preeclampsia.^38^ Compared to the normal control group, patients with gastric cancer expressed significantly lower levels of cMet which decreased with time after diagnosis, indicating that soluble cMet possesses antitumor potential.^39^ In diabetic nephropathy, higher urine cMet levels are correlated with poorer outcomes.^23^ There was also a significant increase in the expression of cMet in patients with AKI.^25^ This is in line with the findings of our study, in which urine cMet levels correlated significantly with proteinuria.

However, rather than leading to disease progression, an increased expression of cMet may induce protection and recovery. Previously, HGF was shown to ameliorate renal damage and reduce acute inflammatory responses in AKI models.^40^, ^41^ Moreover, AKI induction in cMet knockout mice resulted in more severe kidney damage and aggravated apoptosis or inflammatory responses.^42^ The anti‐fibrotic effect of cMet Ab, observed in a unilateral ureteral obstruction model^24^ and ischemic reperfusion injury model,^25^ renal tubular epithelial cells and glomerular endothelial cells^23^ reported recently, also suggests the same.

Our research also has several limitations. First, mesangial cells and IgA isolated from patient serum could not be used in the in vitro experiments. However, we verified that the mesangial cell proliferation and expansion and cMet expression were merged well in the actual IgAN patient; furthermore, the proliferation of mesangial cells and the increase in the level of inflammatory markers induced by TNF‐α may be able to mimic IgAN.^43^, ^44^, ^45^ Second, the sample size was relatively small and a validation in an isolate cohort cannot be obtained; therefore, it is difficult to draw generalized conclusions from this study. In addition, the follow‐up samples of this cohort were not collected, and thus, it was not possible to confirm the changes in ucMet/Cr levels or the effects that the treatment had on the ucMet/Cr levels. Third, the follow‐up was conducted over a relatively short period of time; hence, the long‐term association with ucMet/Cr level was not confirmed. However, since the duration between the initial diagnosis of IgAN and progression to ESRD is prolonged, it is difficult to study ESRD as a clinical outcome. The probability of CR, the clinical outcome of the present study, is a realistic and significant indicator that can be clinically assessed; ucMet/Cr levels can be used to assess this. Lastly, urinary cMet may not be a specific marker of IgAN. We have previously demonstrated the role of urinary cMet or cMet Ab in the context of various diseases such as diabetic nephropathy, unilateral ureteral obstruction and acute kidney injury. Thus, we aimed to emphasize that cMet is not a disease‐specific marker in IgAN patients, but rather has clinical implications and additional effects during diagnosis, treatment and follow‐up in IgAN patients.

In particular, the predictive ability of ucMet/Cr was more significant in the high‐risk group of patients with significant proteinuria. No difference was found in patients with proteinuria <1 g/d. In patients with proteinuria ≥1 g/d, the CR probability decreased with higher ucMet/Cr levels. Therefore, the prognosis can be better predicted by measuring urinary cMet levels in high‐risk patients at the point of initial diagnosis. Furthermore, if cMet Ab is applied to current treatment methods, it could lead to a breakthrough result in the diagnosis and treatment of patients with IgAN. However, studies on a larger scale, including randomized controlled trials and additional mechanistic studies, still need to be conducted.

In conclusion, in the present study, the ucMet levels were consistent with glomerular cMet expression at the time of diagnosis in patients with IgAN. This significantly correlated with proteinuria and could predict CR. Based on the results of the in vitro experiments, we propose that cMet can be used as a marker of survival and recovery, rather than just an indicator of damage. Hence, ucMet may prove to be of clinical significance in patients with IgAN.

## CONFLICT OF INTEREST

The authors confirm that there are no conflicts of interest.

## AUTHOR CONTRIBUTION

**Jung Nam An**: Conceptualization (equal); Data curation (lead); Formal analysis (lead); Visualization (equal); Writing‐original draft (lead); Writing‐review & editing (lead). **Lilin Li**: Investigation (equal); Methodology (equal). **Junghun Lee**: Resources (equal); Supervision (equal); Validation (equal). **Seung‐Shin Yu**: Resources (equal); Supervision (equal); Validation (equal). **Jin Hyuk Kim**: Investigation (lead); Methodology (lead). **Jeonghwan Lee**: Investigation (equal); Methodology (equal). **Yong Chul Kim**: Investigation (equal); Methodology (equal). **Dong Ki Kim**: Supervision (equal); Validation (equal). **Yun Kyu Oh**: Supervision (equal); Validation (equal). **Chun Soo Lim**: Supervision (equal); Validation (equal). **Yon Su Kim**: Supervision (equal); Validation (equal). **Sunyoung Kim**: Resources (equal); Supervision (equal); Validation (equal). **Seung Hee Yang**: Conceptualization (lead); Formal analysis (equal); Investigation (lead); Methodology (lead); Visualization (lead); Writing‐review & editing (equal). **Jung Pyo Lee**: Conceptualization (lead); Funding acquisition (lead); Investigation (equal); Methodology (equal); Project administration (lead); Writing‐original draft (equal); Writing‐review & editing (equal).

## Supporting information

Table S1

## Data Availability

All data generated or analysed in this study are included in this published article (and its supplementary information files Table S1). All other data supporting the presented findings are available from the corresponding author upon request.
