# Supplementary material for: Urinary cMet as a prognostic marker in immunoglobulin A nephropathy
Source: J Cell Mol Med. 2020 Aug 21;24(19):11158–69. doi: 10.1111/jcmm.15636 (PMC7576300; doi:10.1111/jcmm.15636)
Supplement: Supplementary file 1 — Table S1 [file JCMM-24-11158-s001.pdf]

Urinary cMet as a prognostic marker in immunoglobulin A nephropathy

Jung Nam An,[1] Lilin Li,[2,3] Junghun Lee,[4] Seung-Shin Yu,[4] Jin Hyuk Kim,[5] Jeonghwan Lee,[5] Yong Chul Kim,[6] Dong Ki Kim,[2,6] Yun Kyu Oh,[2,5] Chun Soo Lim,[2,5] Yon Su Kim,[2,6] Sunyoung Kim,[4] Seung Hee Yang,[7,8*] Jung Pyo Lee,[2,5*]

[1]Department of Internal Medicine, Hallym University Sacred Heart Hospital, Anyang, Gyeonggi-do, Korea

[2]Department of Internal Medicine, Seoul National University College of Medicine, Seoul, Korea

[3]Department of Intensive Care Unit, Yanbian University Hospital, Jilin, China

[4]R&D Center for Innovative Medicines, Helixmith Co., Ltd., Seoul, Korea

[5]Department of Internal Medicine, Seoul National University Boramae Medical Center, Seoul, Korea

[6]Department of Internal Medicine, Seoul National University Hospital, Seoul, Korea

[7]Seoul National University Kidney Research Institute, Seoul, Korea

[8]Biomedical Research Institute, Seoul National University Hospital, Seoul, Korea

Table S1. Baseline characteristics and demographics according to the groups divided by urine cMet/creatinine level and proteinuria

Table S1. Baseline characteristics and demographics according to the groups divided by urine cMet/creatinine level and proteinuria

		Group 1 (n = 42)	Group 2 (n = 15)	Group 3 (n = 62)	Group 4 (n = 55)	*P* value
**Age (years)**		31 (20, 46)	36 (23, 49)	43 (35, 53)	45 (33, 60)	0.002
**Male gender**		31 (73.8)	7 (46.7)	33 (53.2)	20 (36.4)	0.009
**Smoking history**		5 (11.9)	1 (6.7)	13 (21.0)	11 (20.0)	0.406
**Diabetes Mellitus**		1 (2.4)	0 (0.0)	2 (3.2)	4 (7.3)	0.204
**Hypertension**		15 (35.7)	5 (33.3)	39 (62.9)	40 (72.7)	0.001
**Systolic Blood Pressure (mmHg)**		122.8 ± 16.1	121.5 ± 13.0	127.3 ± 19.5	129.0 ± 19.3	0.270
**Diastolic Blood Pressure (mmHg)**		76.1 ± 11.7	74.2 ± 9.4	80.9 ± 13.3	81.5 ± 15.7	0.079
**Body mass index (kg/m^2)**		23.6 ± 3.3	23.3 ± 4.0	24.2 ± 3.5	24.0 ± 3.2	0.681
**Microscopic hematuria**		39 (92.9)	14 (93.3)	56 (90.3)	51 (92.7)	0.895
**SMK Lee grade**	I	4 (9.5)	0 (0.0)	0 (0.0)	1 (1.8)	<0.001
	II	28 (66.7)	8 (53.3)	28 (45.2)	20 (36.4)	
	III	9 (21.4)	4 (26.7)	17 (27.4)	15 (27.3)	
	IV	0 (0.0)	0 (0.0)	6 (9.7)	7 (12.7)	
	V	0 (0.0)	0 (0.0)	4 (6.5)	3 (5.4)	
**Haas Class**	I	3 (7.1)	0 (0.0)	0 (0.0)	0 (0.0)	0.001
	II	4 (9.5)	1 (6.7)	8 (12.9)	4 (7.3)	
	III	25 (59.5)	7 (46.7)	19 (30.6)	16 (29.1)	
	IV	8 (19.0)	4 (26.7)	19 (30.6)	18 (32.7)	
	V	0 (0.0)	0 (0.0)	9 (14.5)	8 (14.5)	
	VI	1 (2.4)	0 (0.0)	0 (0.0)	0 (0.0)	
**Mesangial hypercellularity**		42 (100.0)	14 (93.3)	58 (93.5)	52 (94.5)	0.204
**Interstitial fibrosis/tubular atrophy**		36 (85.7)	8 (53.3)	56 (90.3)	50 (90.9)	0.001
Moderate to severe		7 (16.7)	1 (6.7)	18 (29.0)	14 (25.5)	0.063
**Interstitial inflammation**		30 (71.4)	9 (60.0)	52 (83.9)	51 (92.7)	0.006
Moderate to severe		3 (7.1)	1 (6.7)	15 (24.2)	12 (21.8)	0.006
**Vessel**						

Fibrointimal thickening	7 (16.7)	5 (33.3)	25 (40.3)	25 (45.5)	0.023
Hyaline arteriolosclerosis	2 (4.8)	1 (6.7)	14 (22.6)	11 (20.0)	0.057
**Global sclerosis (%)**	8.3 (0.0, 21.2)	2.6 (0.0, 15.4)	28.6 (11.7, 46.2)	20.0 (5.3, 41.2)	<0.001
**Segmental sclerosis (%)**	0.0 (0.0, 6.9)	0.0 (0.0, 7.4)	6.2 (0.0, 18.7)	5.9 (0.0, 15.8)	0.005
**Crescent (%)**	0.0 (0.0, 0.0)	0.0 (0.0, 3.2)	0.0 (0.0, 0.0)	0.0 (0.0, 0.0)	0.471
**Laboratory findings**					
Serum creatinine (sCr) (mg/dL)	0.98 (0.80, 1.15)	0.72 (0.60, 0.88)	1.28 (0.85, 1.62)	1.05 (0.79, 1.45)	<0.001
Estimated GFR (mL/min/1.73 m^2)	89.8 (64.5, 109.4)	113.4 (88.4, 132.3)	55.1 (42.3, 84.2)	62.2 (48.3, 87.6)	<0.001
Urine protein/creatinine ratio (mg/mgCr)	0.31 (0.17, 0.64)	0.75 (0.30, 0.89)	2.06 (1.48, 2.75)	3.26 (2.02, 4.37)	<0.001
Immunoglobulin A (mg/dL)	322.0 (227.0, 404.5)	315.0 (269.0, 395.0)	321.0 (251.0, 411.0)	339.0 (252.5, 462.5)	0.613
Albumin (g/dL)	4.1 (4.0, 4.3)	4.0 (3.8, 4.2)	3.7 (3.5, 4.0)	3.6 (3.3, 3.8)	<0.001
hs-CRP (mg/dL)	0.06 (0.03, 0.17)	0.05 (0.02, 0.35)	0.10 (0.04, 0.24)	0.12 (0.04, 0.43)	0.300
Total cholesterol (mg/dL)	160.0 (139.5, 190.0)	178.0 (162.0, 211.0)	205.0 (159.8, 233.0)	194.0 (165.5, 215.5)	0.001
Uric acid (mg/dL)	6.2 (5.0, 7.2)	4.6 (4.0, 6.1)	6.9 (5.4, 8.1)	6.1 (5.2, 7.5)	0.022
**Urine cMet (ng/mL)**	0.22 (0.00, 0.79)	1.62 (1.20, 2.36)	0.16 (0.00, 0.58)	1.97 (1.39, 3.31)	<0.001
**Urine cMet/Cr (ng/mgCr)**	0.002 (0.000, 0.006)	0.018 (0.015, 0.022)	0.001 (0.000, 0.007)	0.023 (0.019, 0.045)	<0.001
**Treated with RAS blockade**	20 (47.6)	7 (46.7)	38 (61.3)	41 (74.5)	0.034
**Treated with statin**	8 (19.0)	1 (6.7)	24 (38.7)	25 (45.5)	0.004
**Treated with immunosuppressive agents**	0 (0.0)	0 (0.0)	11 (17.7)	15 (27.3)	0.001

The data are expressed as the proportion (%), mean ± SD or median (IQR).

Abbreviations: GFR, glomerular filtration rate; hs-CRP, high-sensitivity C-reactive protein; RAS, renin-angiotensin system
